# Role of interleukin‐15 in cardiovascular diseases

**DOI:** 10.1111/jcmm.15296

**Published:** 2020-05-14

**Authors:** Lei Guo, Ming‐fei Liu, Ji‐niu Huang, Jia‐min Li, Jun Jiang, Jian‐an Wang

**Affiliations:** ^1^ Department of Cardiology The Second Affiliated Hospital Zhejiang University School of Medicine Hangzhou China; ^2^ Cardiovascular Key Lab of Zhejiang Province Hangzhou China

**Keywords:** cardiovascular diseases, inflammation, interleukin‐15, interleukin‐15 receptor

## Abstract

Interleukin (IL)‐15 is a recently identified cytokine, which belongs to the interleukin‐2(IL‐2) family, and plays an important role in innate and adaptive immunoreaction. Given the fact that the structure of IL‐15 is partially similar to IL‐2, they share some common biological effects, including immunoregulation. IL‐2 was proven to protect cardiac function in mouse myocardial infarction models. Cardiovascular diseases (CVDs) dominate the cause of mortality worldwide. Besides atherosclerosis, inflammation is also widely involved in the pathogenesis of many CVDs including hypertension, heart failure (HF) and aneurysm. IL‐15, as a pro‐inflammatory cytokine, is up‐regulated in some cardiovascular diseases, such as myocardial infarction and atherosclerosis. The current understanding of IL‐15, including its signal pathway and cellular function, was described. Furthermore, IL‐15 has a protective effect in myocardial infarction and myocarditis by decreasing cardiomyocyte death and improving heart function. The inhibited effect of IL‐15 in ductus arteriosus (DA) should be focused on. IL‐15 promoted atherogenesis. IL‐15 may be a good target in treatment of cardiovascular diabetology. Finally, future research direction of IL‐15 deserves attention. Since IL‐15 plays several roles in CVDs, understanding the role of the IL‐15/IL‐15R system may provide a scientific basis for the development of new approaches that use IL‐15 for the treatment of CVDs.

## 1 INTRODUCTION

Interleukin 15 (IL‐15) is a recently identified cytokine widely distributed in various tissue types, including heart, lung, liver, thymus and kidney, which is a critical mediator of inflammation.^1^ It has been well recognized that inflammation actively involved in the development and progression of cardiovascular diseases (CVDs), in both temporal and spatial aspects.^2^ An appropriately regulated inflammatory response can help the cardiovascular system to adjust under different stimuli and preserve its function; however, systemic inflammatory response could be detrimental and usually lead to impaired repair process or poor remodelling. Inflammatory factors, such as C‐reactive protein (CRP), tumour necrosis factors (TNFs), interferons (IFNs) and interleukins (ILs), are the most common mediators in inflammatory responses, and have been applied in disease diagnosis and staging, prognosis estimation and applied in therapeutic intervention.^3^ Up‐regulated IL‐15 levels were found in both human and animal atherosclerotic lesions.^4^, ^5^, ^6^

### 1.1 Association between CVDs and Inflammation

CVDs are the world's leading cause of mortality. Various risk factors entailing elevated cholesterol levels, insulin resistance, diabetes and obesity are involved in the initiation and development of atherosclerotic lesions.^6^ Heart failure (HF) may be the terminal stage of various cardiovascular diseases, as one of major obstacles to CVDs management in the 21st century. Over the past few years, the prevalence of HF has been on the rise, mainly due to the ageing population trend, and an increase in patients with CVDs surviving with the help of modern interventional strategies.^7^ In Abeywardena et al 2009, the data gathered about CVDs were shown to demonstrate that systemic inflammation is frequently found in CVDs. Inflammation leads to elevated levels of the pro‐inflammatory cytokine that disrupt the systematic balance, suggesting that inflammation may lead to the damage and dysfunction of the cardiovascular system, such as interleukin‐6(IL‐6) and interleukin‐1β(IL‐1β). Acute myocardial infarction has been reported to be associated with the production of pro‐inflammatory cytokines with high concentrations of IL‐1β, tumour necrosis factor(TNF‐α) and IL‐6.^8^ Biological activities of inflammatory factors in CVDs are effective in the plaque formation and rupture, endothelial dysfunction, and eventually, coronary thrombosis.^9^ ILs are a family of cytokines that exert their effects via gene activation involved in differentiation, cellular stimulation, growth, cellular effector function and functional cell surface receptor expression.^10^

### 1.2 IL‐15, a member of IL‐2 family, is a crucial mediator of inflammation

**Figure 1:**
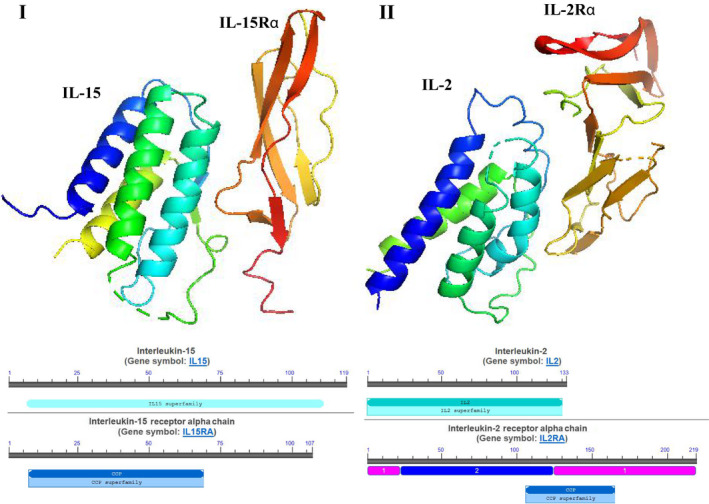
The three‐dimensional structure of IL‐15 and IL‐2. (I) IL‐15 and IL‐15Rα. (II)IL‐2 and IL‐2Rα. PyMOL (version 0.99; DeLano Scientific, San Carlos, CA, USA) was used to view the graphic.

**Figure 2:**
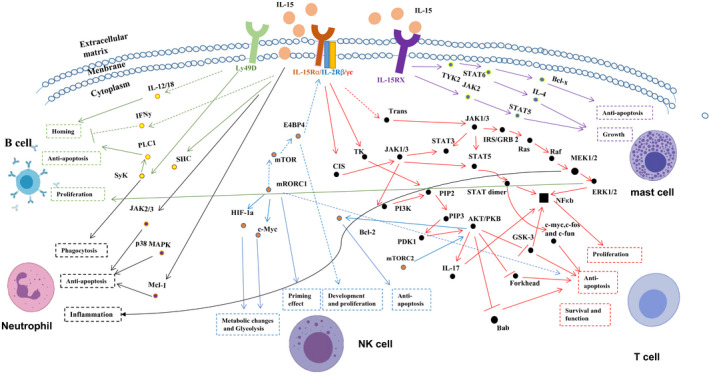
Signal transduction by IL‐15. IL‐15 acts differently on various cell types through a number of pathway and signal molecules. (The data are from NCBI and KEGG)

Firstly, IL‐15 was discovered in the field of haematology as a member of the immunoregulatory cytokines family with multiple functions, including dictating T‐cell response, regulating tissue repair and B‐cell homing, modulating inflammation and activating NK cells.^11^ Subsequently, it was found to be expressed by monocytes, macrophages and other cell types involved in immune regulation comprising both innate and adaptive immune responses.^5^ IL‐2 and IL‐15 bind to a common heterodimeric receptor composed of the IL‐2/15Rβ (CD122) and common γ (gc or CD132) chains for signal transmission. The specific activity of both cytokines is conferred by the corresponding alpha receptor chains, IL‐2Rα(CD25) and IL‐15Rα(CD215).^12^ IL‐15Rα binds IL‐15 with high affinity (Kd = 100 pM), whereas isolated IL‐2Rα is a low‐affinity receptor for IL‐2 (Kd = 10 nM). IL‐15 and IL‐2 are similar in three‐dimensional structure (Figure 1).^13^ The IL‐15 gene is located on the human chromosome 4q31 and the central region of mouse chromosome 8.^14^ Both humans and laboratory mice exhibit detectable levels of IL‐15 in the circulation, suggesting that it can exert endocrine effects on the cell types which do not express the cytokine itself.^15^ IL‐15Rα consists of a 173‐amino acid (aa) extracellular domain, a single 21‐aa membrane‐spanning region and a 37‐aa cytoplasmic domain. This is structurally similar to IL‐2Rα with a conserved extracellular protein‐binding Sushi domain. Also, the intron‐exon organization of the IL‐15Rα gene is similar to that of IL‐2Rα. Despite these similarities, IL‐15Rα shares little sequence homology to IL‐2Rα.^16^ IL‐15Rα is widely expressed in humans and mice independently of IL‐2R/IL‐15Rβ‐γ chain. It binds to IL‐15 with a high affinity and retains IL‐15 on the cell surface. IL‐15 bound to IL‐15Rα can also recycle through endosomal vesicles for many days (endosomal recycling), resulting in the persistence of membrane‐bound IL‐15.^17^ Up‐regulation of IL‐15 and IL‐15Rα is found during inflammatory responses. IL‐15 is not a simple growth factor, but also a multifaceted regulator. In addition to it, the signalling pathways of IL‐15 are highly connected with various cell types, which are mediated through numerous pathways and signalling molecules (Figure 2).^18^ Accumulating evidence suggests that skeletal muscles are also a major source of this cytokine.^19^, ^20^, ^21^ The expression of IL‐15 and IL‐15Rα has been detected in many cell types and is summarized in Table 1.^22^, ^23^, ^24^, ^25^, ^26^

**Table 1:** IL‐15’s expression in many types of cells

Types of cells	IL‐15	IL‐15 receptor	References
Cardiomyocytes	+	+	^27^
Fibroblasts	+	+	^28^
Muscle cells	+	Unknown	^10^
Bone marrow stromal cells	+	Unknown	^26^
NK cells	‐	+	^26^
DC cells	+	+	^25^
Neutrophils	+	+	^22^
Macrophages	+	+	^23^
Monocytes	+	+	^24^
B cells	Unknown	+	^28^
T cells	+	+	^28^
Endothelial cells	+	Unknown	22, 28
Renal epithelial cells	+	Unknown	26, 28
Adipocytes	+	Unknown	^26^

## 2 ROLE OF IL‐2 IN CVDS

In Dinh et al 2012, the data gathered about IL‐2 were shown to demonstrate that the IL‐2 complex which comprises IL‐2 and anti‐IL‐2 monoclonal antibody (mAb)(JES6‐1) selectively expands Treg cells up to six folds and effectively inhibits the development and progression of atherosclerosis in ApoE ‐/‐ mice significantly attenuates ventricular remodelling, resulting in reduced infarct size and improved left ventricular (LV) function.^27^ Moreover, in Webster et al 2009, the data demonstrated that IL‐2 attenuated cardiomyocyte apoptosis in a mouse myocardial infarction model.^28^ In Zeng et al 2016, the data demonstrated that the IL‐2 complex exerts protective effects by directly inhibiting the infiltration of inflammatory macrophages and facilitating the polarization of anti‐inflammatory M2 macrophages, which in turn, attenuates the apoptosis of cardiomyocytes and the local inflammatory responses. The expansion of Treg cells by the IL‐2 complex may be a potentially valuable approach in improving ischaemic heart disease.^29^ Bouchentouf et al 2011 showed that a single intravascular injection of recombinant human IL‐2(rhIL‐2) two days following myocardial infarction (MI) improved the left ventricular (LV) ejection fraction by 27.7% of immune competent mice but did not affect the cardiac function of the immunodeficient (NOD‐SCID IL2Rα null) mice. The immunohistochemical analysis of infarcted heart in mice showed that collagen deposition decreased and that capillary density increased in the scar and border areas of infarcted hearts, respectively following rhIL‐2 injection. Cardiac angiogenesis is enhanced by the administration of rhIL‐2 and helps preserve the integrity and function of myocardium.^30^ Since two subunits of IL‐15 receptors and IL‐2 receptors are the same, the biological activities of IL‐15 might partially overlap with those of IL‐2. Increased IL‐15 levels have been observed in the cardiovascular patients, and IL‐15 immunoreactivity has been detected in vulnerable atherosclerotic plaques. Moreover, IL‐15 is expressed by inflammatory cells localized in the vulnerable atherosclerotic plaques, and serum IL‐15 concentration is significantly higher in patients with CAD or peripheral artery disease than in healthy individuals.^6^, ^31^ In the light of these findings, it is reasonable to speculate that IL‐15 might also play a role in the cardiovascular system. The correlation between IL‐15 levels and numerous CVDs is shown in Table 2.

**Table 2:** Levels of IL‐15 in cardiovascular diseases

Disease types	IL‐15's level	References
1. Acute heart disease compared with chronic heart disease patient 2. Chronic heart disease compared with control 3. Peripheral artery disease compared with control	Significantly higher (serum levels)	^6^
Myocardial infarction	(4‐6h) after onset of ischaemia: +++ >6‐8h from the onset of ischaemic symptoms and signs: + (frozen cardiac tissue's WB)	^3^
Atherosclerosis hypercholesterolemia	Significantly higher (spleen and blood)	^34^
CCAD compared with non‐CAD	IL‐15 and IL‐15Rα are significantly higher (Plasma levels)	^5^

## 3 ROLE OF IL‐15 IN CVDS

### 3.1 Role of IL‐15 in myocarditis and oxidative stress

**Figure 3:**
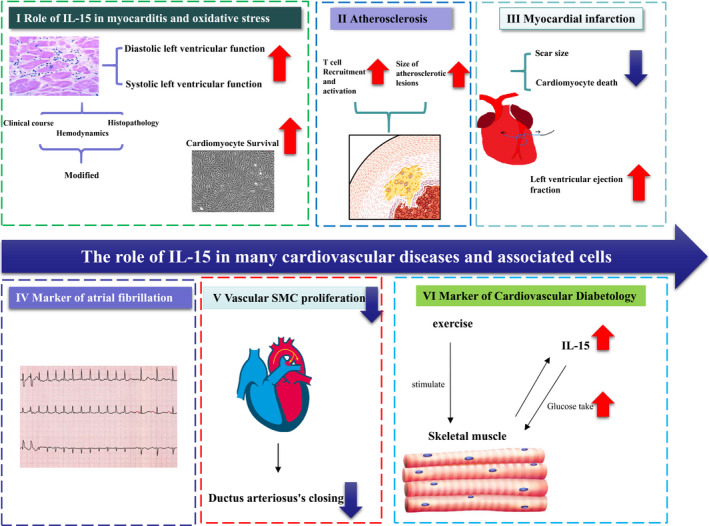
The role of IL‐15 in many cardiovascular diseases and associated cells. (I) Role of IL‐15 in myocarditis and oxidative stress (II) Atherosclerosis (III) myocardial infarction (IV) Marker of atrial fibrillation (V) Ductus arteriosus (VI) Marker of cardiovascular diabetology.

Bigalke et al 2009 demonstrated that treatment with IL‐15 exerted a positive effect on CVB3‐induced murine myocarditis, an animal model of virus‐induced myocarditis, haemodynamics, and the histopathology of virus‐induced myocarditis. After treatment with IL‐15, the body and heart weight of infected animals are normalized and the systolic and diastolic left ventricular functions are significantly improved as compared to that of the untreated animals.^32^ These phenotypes may be associated with the effect of IL‐15 on cardiomyocytes. In addition, IL‐15 receptors have been identified on the surface of cardiomyocytes that showed the signalling system to protect the hypoxic cardiomyocytes from cellular death and rescue from oxidative stress post‐IL‐15 treatment (Figure 3 I). Herein, in Yeghizarians et al 2014, the data gathered about myocarditis were shown to demonstrate that IL‐15 and its receptors are present on the cardiomyocytes. Furthermore, the administration of supplemental IL‐15 increased the survival of cardiomyocytes under oxidative stress. While under oxidative stress, it promoted the survival of cardiomyocytes via STAT3 phosphorylation. Also, IL‐15 protected the cardiomyocytes from oxidative stress through the PI3K/ERK1/2 pathway.^33^

### 3.2 Role of IL‐15 in atherosclerosis

In Wuttge et al 2001, IL‐15 was found to be up‐regulated in both human and animal atherosclerotic lesions and may contribute to the recruitment and the activation of T cells during atherogenesis.^4^ IL‐15 is up‐regulated in hypercholesterolemic mice.^34^ Vaccination against IL‐15 was accomplished by oral administration of live attenuated *Salmonella typhimurium* transformed with a eukaryotic expression vector encoding IL‐15. This vaccination method induced a robust, IL‐15‐specific, cytotoxic immune response, resulting in the killing of cells overexpressing IL‐15. Moreover, Fisman et al (2008) demonstrated that the vaccination against IL‐15 also reduced the size of atherosclerotic lesions in hypercholesterolemic low‐density lipoprotein receptor knock‐out mice (LDLr‐/‐ mice)^35^ (Figure 3 II). IL‐15 is also involved in the expansion and survival of natural killer T (NKT) cells, which can form an essential link between the innate and adaptive immune responses and enhance atherosclerosis.^36^ Increased IL‐15 levels have been observed in cardiovascular patients where immunoreactivity has been detected within the vulnerable atherosclerotic plaques. Moreover, IL‐15 is expressed by inflammatory cells localized to vulnerable atherosclerotic plaques and serum IL‐15 concentration is significantly higher in patients with CADs or peripheral artery disease than in healthy individuals.^6^, ^37^ These observations may be connected with the effect of IL‐15 in obesity. Obesity status of the patients may be involved in increasing IL‐15 expression. Furthermore, the adipose tissue develops an inflammatory environment due to infiltrating macrophages which are a source of numerous pro‐inflammatory cytokines. Macrophages are actively involved in atherogenesis.^38^, ^39^ Obese individuals have lower levels of circulating IL‐15 in blood, while higher levels of IL‐15 in individuals were associated with a lean body type.^40^ Mice with targeted deletion of *IL15* (IL‐15KO mice) exhibited higher amounts of body fat than control mice. Interestingly, IL‐15 is a cytokine that inhibits lipid deposition in cultured adipocytes and decreases the deposition of adipose tissue in laboratory rodents. In addition, a model with genetic variation in IL15Rα modulates the activity and bioavailability of the cytokine, which in turn, regulates adiposity.^1^, ^20^, ^41^ The exact association between IL‐15 and atherosclerosis needs further elucidation.

### 3.3 Role of IL‐15 in myocardial infarction

Coronary artery disease may lead to myocardial infarction (MI), a leading cause of death worldwide.^42^ In Ameri et al 2020, the data gathered about myocardial infarction showed that administration of IL‐15 could improve heart function of C57/B6 mouse after myocardial infarction through decreased cardiomyocyte death, decreased scar size and improved vascularity (Figure 3 III). It suggests that IL‐15 plays a positive role in myocardial infarction.^43^ This antifibrotic role is consistent with another study. In Wuttge et al 2010, the data showed that IL‐15 may prevent myofibroblast differentiation in human foetal lung fibroblast via antagonizing TGF‐β1‐mediated SMAD2/3 signalling cascades.^44^ In Dozio et al 2014, the data showed that the plasma IL‐15 and IL‐15Rα levels were higher in coronary artery diseases (CADs) than non‐CADs patients.^5^ While the levels of IL‐15, high sensitive C‐reactive protein(hs‐CRP), blood urea nitrogen(BUN), total cholesterol(TC), triglyceride(TG), and high‐density lipoprotein cholesterol (HDL‐C)and body mass index(BMI)were associated with CHD in univariate analysis, only IL‐15 and hs‐CRP levels were associated with CADs in multivariate logistic regression analysis. ^45^

### 3.4 IL‐15 acts as a biomarker of atrial fibrillation (AF)

Chronic inflammation due to autoimmune diseases is associated with a high rate of supraventricular and ventricular arrhythmias. Minimizing the inflammatory burden by controlling the disease activity may help in reducing the arrhythmic burden.^30^ AF is the most common arrhythmia in a clinical setting. Accumulating evidence implicates an association between inflammation and AF. In Borowiec et al 2015, a previous study included 158 consecutive patients that opposed to sporadic cases with refractory episodes of AF and without structural heart disease. Basic inflammatory markers remained unchanged in patients with refractory AF episodes in the prospective 1‐year observation except for IL‐15, which was correlated to the numbers of AF episodes, and hence, could be applied in disease prognosis^46^ (Figure 3 IV).

### 3.5 Role of IL‐15 in ductus arteriosus (DA)

DA is a foetal arterial connection between the main pulmonary artery and the descending aorta that closes immediately after birth. During the first few hours after birth in the newborns, an acute and functional closure is observed as a result of the smooth muscle contraction of the DA, which is triggered by an increase in oxygen tension and a decline in circulating prostaglandin E (PGE2).^47^ The neointimal cushion of DA is formed by several cellular processes, including smooth muscle cells (SMCs) migration and proliferation, extracellular matrix production under the endothelial layer and decreased assembly of the elastin fibre.^48^, ^49^ Neointimal cushion formation is an important vascular reconstruction closure. The inflammatory response to vascular injury or atherosclerosis may involve the pathogenesis of NCF. In Iwasaki et al 2007, the data showed that IL‐15 mRNA expression in rat DA was significantly higher than in the aorta. The immunohistochemistry results showed the localization of IL‐15 in DA. IL‐15 immunoreactivity was mainly detected in the internal elastic laminae of rat DA by IL‐15 antibody, but less in SMCs. CX3CR1 chemokine signalling was involved in atherogenesis and promotes SMC proliferation, but IL‐15 significantly decreased the expression of CX3CR1 in DA SMCs. Also, IL‐15 significantly attenuated the platelet‐derived growth factor (PDGF‐BB)‐mediated SMC proliferation. It inhibited physiologic vascular remodelling process during the DA (Figure 3 V). And it contributed to pathogenesis of persistent DA.^50^ Down‐regulation of the expression of IL‐15 may benefit the closure of DA.

### 3.6 Role of IL‐15 in glycometabolism

IL‐15 is not only a pro‐inflammatory cytokine but also a ‘myokine’. Myokine is a kind of cytokine secreted by skeletal muscle, which has been identified as an endocrine organ. Besides IL‐15, there are many myokines, such as IL‐6, irisin and decorin.^51^ Skeletal muscle contractile activity increases the production of myokines. And these myokines can stimulate glucose transport.^52^ In Yang et al 2013, the data showed that high‐fat diet caused down‐regulation of IL‐15 in muscle and IL‐15R in adipose tissue. But exercise training can improve obesity and reverse this down‐regulation.^53^ In Yargic et al 2019, the data showed that serum IL‐15 levels increased after an acute endurance exercise.^54^ All mentioned above is about the production of IL‐15. IL‐15 also plays some roles in skeletal muscle and metabolism. It exerts an anabolic effect in skeletal muscle by decreasing proteolytic rate at the cellular level.^55^ Moreover, it increases glucose uptake in skeletal muscle at the cellular level. And IL‐15 might be an antidiabetogenic drug in the future.^56^ Some researchers found that overexpression of IL‐15 in mouse skeletal muscle can improve glucose metabolism in skeletal muscle through AMPK pathway.^57^, ^58^ Others found that it is Jak3/STAT3 pathway that regulates glucose metabolism.^59^ In Quinn et al 2011, they found that overexpression of IL‐15 in mouse promoted resistance to obesity and increased insulin sensitivity.^60^ IL‐15 is one of biomarkers in cardiovascular diabetology^61^ (Figure 3 VI). IL‐15 may be a good target in treatment of type 2 diabetes mellitus.^62^

## 4 DISCUSSION

It is not hard to draw a conclusion that IL‐15 benefits cardiovascular system. Some phenomena about IL‐15 have been found, but the mechanism needs further study. IL‐15 has a positive effect on CVB3‐ induced murine myocarditis and promotes survival in cardiomyocytes.^21^ Moreover, administration of IL‐15 could improve heart function of C57/B6 mouse after myocardial infarction by reducing cardiomyocyte death.^43^ It is interesting that the IL‐15 level increases in patients with CVDs. Plasma levels of IL‐15 and IL‐15Rα were higher in CAD than non‐CAD patients.^3^, ^5^, ^6^, ^34^ It also aggravates the development of atherosclerotic lesions in LDL‐R‐deficient mice.^35^ But IL‐15 inhibits the deposition of lipids in cultured adipocytes and decreases the deposition of adipose tissues in laboratory rodents.^37^, ^41^ The roles of IL‐15 in hyperlipidaemia and atherosclerosis are controversial, and the underlying mechanism needs to be further explored. The cytokine is also deemed as a marker of refractory atrial fibrillation.^46^ Although the potential protective role of IL‐15 has been demonstrated in numerous in vitro and in vivo models of cardiovascular diseases, further studies are necessary to confirm the accurate mechanism and the most effective administration of IL‐15 activators and inhibitors in these diseases. The evidence so far suggests that the association between signalling pathways and IL‐15 in the cardiovascular system involves JAK/STAT and SMAD2/3 signalling; however, further research is needed.^33^, ^34^ IL‐15 regulated glucose metabolism and may be a good target in treatment of cardiovascular diabetology.^62^ Since IL‐15 plays several roles in CVDs, understanding the role of the IL‐15/IL‐15R system may provide a scientific basis for the development of new approaches that use IL‐15 for the treatment of CVDs.

## CONFLICT OF INTEREST

The authors declare that they have no competing interests.

## AUTHOR CONTRIBUTION

Lei Guo wrote the paper and drew the graphic, and Ming‐fei Liu analysed the data and wrote the paper. Ji‐niu Huang collected the literature. Jia‐min Li analysed the data. Jun Jiang designed the research and drew the graphic. Jian‐an Wang designed the research and drew. All authors read and approved the final manuscript.

## Data Availability

The NCBI database accession number for the protein profile data of IL‐15 and IL‐2 reported in this article is (PBD ID:1Z92, PBD ID:2Z3R).
